# Alpha-synuclein at the crossroads of host–virus interactions: immunological roles beyond the nervous system

**DOI:** 10.1128/jvi.00191-26

**Published:** 2026-06-09

**Authors:** Valentina Artusa, Fiona Limanaqi, Elena Santacroce, Mario Clerici, Andrea Cossarizza, Mara Biasin, Lara Gibellini, Daria Trabattoni

**Affiliations:** 1 Department of Biomedical and Clinical Sciences, University of Milan, Milan, Italy; 2 Department of Medical and Surgical Sciences for Children and Adults, University of Modena and Reggio Emilia, Modena, Italy; 3 Department of Pathophysiology and Transplantation, University of Milan, Milan, Italy; 4 IRCCS Fondazione Don Carlo Gnocchi ONLUS, Milan, Italy; Universiteit Gent, Merelbeke, Belgium

**Keywords:** infection, proteostasis, antigen presentation, inflammation, neurodegeneration

## Abstract

Alpha-synuclein (α-syn) is best known as a presynaptic protein that supports synaptic vesicle dynamics and neurotransmission. Conversely, misfolded or aggregated α-syn represents a hallmark of synucleinopathies, including Parkinson’s disease. Beyond the nervous system, α-syn has been detected in peripheral compartments, including blood cells and selected epithelial tissues, although the robustness and context dependence of expression outside neuronal and erythroid lineages remain under active investigation. Also, it can be released extracellularly through unconventional secretion or cell damage. These observations have reframed α-syn as an immune-relevant molecule positioned at host–pathogen interfaces, endowed with antimicrobial peptide-like and damage-associated molecular pattern-like properties that enable shaping of both innate and adaptive immunity. Increasing evidence indicates that viral challenge alters α-syn expression, localization, and conformational states in central and peripheral settings, in part through interferon-dependent programs that couple antiviral immunity with cellular homeostasis. A plethora of RNA viruses, such as influenza virus, flavivirus, enterovirus, and coronavirus, perturb α-syn abundance, post-translational modifications, trafficking, secretion, and aggregation propensity. These effects converge on shared mechanisms that include altered proteostasis, autophagy–lysosomal dysfunction, oxidative and mitochondrial injury, and inflammatory signaling. Importantly, outcomes are highly context dependent, ranging from cell-intrinsic antiviral restriction to aggregation-prone states that may fuel chronic inflammation and neurodegeneration. Collectively, the evidence discussed herein supports a dual framework in which α-syn contributes to antiviral defense; yet, under conditions of sustained inflammation or impaired clearance, it may undergo pathological transformation that promotes neuronal damage. Defining when virus-induced α-syn responses are protective versus pathogenic, and clarifying their relevance to human disease, will be critical for developing strategies that target host–virus interactions, neuroinflammation, and α-syn proteostasis in infection-associated synucleinopathies.

## INTRODUCTION

### Classical functions of α-Synuclein: from synaptic homeostasis to Parkinson’s disease pathology

Alpha-synuclein (hereafter, abbreviated as α-syn) is a small, intrinsically disordered protein predominantly localized at presynaptic terminals in the central nervous system (CNS). Endowed with structural plasticity, it is involved in key aspects of synaptic physiology, including regulation of synaptic vesicle trafficking, modulation of neurotransmitter release, and maintenance of synaptic plasticity. In detail, α-syn acts as a molecular chaperone for SNARE complex assembly, thereby facilitating efficient neurotransmission and contributing to the fine-tuning of neuronal connectivity (1). Although its mechanisms of action remain to be fully elucidated, the physiological presence of α-syn is generally regarded as essential for synaptic homeostasis and normal neuronal function (2). The same structural and functional properties that enable α-syn to support neurotransmission, however, may also render it vulnerable to maladaptive conformational changes, thereby linking its normal biology to potential pathogenic processes. Indeed, the pathological significance of α-syn has been firmly established in Parkinson’s disease (PD) and related synucleinopathies (2). Misfolding and aggregation of α-syn lead to the formation of insoluble fibrillar inclusions known as Lewy bodies and Lewy neurites, which are pathological hallmarks of PD. Lewy bodies and Lewy neurites are intracellular inclusions enriched in aggregated α-syn species, frequently phosphorylated at Ser129 (pSer129 α-syn), together with other proteins and membranous components, and are widely used as neuropathological correlates of synucleinopathy. These aggregates disrupt cellular proteostasis, impair mitochondrial and lysosomal function, and ultimately contribute to neurodegeneration. Moreover, abnormal α-syn species are thought to propagate in a prion-like manner across neuronal and systemic networks, thereby driving disease progression and accounting for the stereotypical spatiotemporal distribution of pathology in PD. Thus, α-syn embodies a paradoxical duality, functioning as a critical regulator of synaptic biology under physiological conditions, while acting as a central pathogenic effector in the context of neurodegenerative disease (3).

Although initially regarded as a neuron-specific protein, accumulating evidence indicates that α-syn is present beyond the CNS, being detected in the enteric nervous system and in blood-derived compartments, particularly erythrocytes and platelets (4–8). Reports of α-syn detection in additional peripheral tissues, including epithelial compartments, support a broader distribution that may be context- and method-dependent (4–8). The detection of α-syn in these peripheral compartments is especially relevant from an immunological perspective, as it positions this protein at critical interfaces of host defense and systemic inflammation, thereby providing a mechanistic link to its emerging role in immune regulation and pathogen response. Therefore, the presence of α-syn in peripheral tissues has stimulated growing interest in its potential roles in systemic physiology and in pathological processes outside the brain. Its induction in response to cellular stressors, including microbial products and inflammatory stimuli, supports the hypothesis that α-syn functions as an inducible mediator at the interface of the nervous and immune systems (9). Furthermore, extracellular α-syn, released through unconventional secretion or following cell damage, can act as a signaling molecule capable of modulating innate and adaptive immune responses (10–13). These findings point to a broader conceptual framework in which α-syn should not only be viewed as a neuronal protein implicated in neurodegeneration but also as a multifunctional effector engaged in immune surveillance and potentially in responses to infectious challenges. Notably, recent reports suggest that viral infections represent a particularly potent trigger for α-syn induction and redistribution, raising the possibility that this protein contributes directly to host–virus interactions by coupling neuronal stress responses with antiviral immunity (14–16).

### α-Synuclein as a potential mediator of host–virus interactions

Viral infections impose strong selective pressures on neuronal and peripheral systems, often triggering innate immune responses and altering proteostatic balance. Experimental studies have shown that α-syn expression can be induced upon viral challenge in both central and peripheral tissues, suggesting that this protein may participate in antiviral defense (15, 17, 18). The induction of α-syn, together with its ability to modulate inflammatory signaling and interact with immune receptors, suggests that α-syn could be a molecular mediator in neuroimmune responses to viral pathogens. Indeed, several lines of evidence linking viral infections to the initiation or exacerbation of neurodegenerative processes, including PD, are present and have recently been reviewed and summarized in the literature (19). Viral pathogens have been implicated as potential environmental triggers that may accelerate the pathological transformation of α-syn, fostering its abnormal conformations, accumulation, and intercellular dissemination, thereby bridging infectious episodes with long-term neuropathological consequences. Exploring the role of α-syn in viral infections is therefore of dual importance: first, to understand its potential protective or immunomodulatory functions in acute host defense, and second, to elucidate whether infection-driven alterations of α-syn biology contribute to chronic inflammation and the pathogenesis of synucleinopathies. This dual perspective frames α-syn as a pivotal molecule at the intersection of virology, immunology, and neurodegeneration. Importantly, the emerging view of α-syn as an immune-relevant molecule is consistent with evidence that this protein participates in stress- and proteostasis-related programs in non-neuronal settings, including myeloid and tumor cells (20, 21), reinforcing its pleiotropic nature and the likelihood that infection-induced α-syn responses reflect a systemic, multi-tissue biology.

## α-SYNUCLEIN: BEYOND SYNAPSES—AN IMMUNE PLAYER

### α-Synuclein as an antimicrobial peptide: evidence and implications

A growing body of evidence supports the hypothesis that α-syn may act as an antimicrobial peptide (AMP), conferring protection against invading pathogens. Like classical AMPs, α-syn possesses intrinsic structural features that enable interactions with microbial membranes. Its amphipathic N-terminal domain allows insertion into lipid bilayers, where it can destabilize membrane integrity and impair microbial viability (22). Experimental studies have demonstrated that α-syn exhibits direct antimicrobial activity against a range of bacterial (23) and viral pathogens (17, 18), suggesting that its evolutionary conservation may, at least in part, reflect a role in host defense. Notably, the antiviral effects described in neuronal systems suggest that α-syn induction may participate in early virus-restriction mechanisms that interface with interferon-dependent signaling pathways, a concept further developed in later sections.

Beyond its direct microbicidal effects, α-syn may also exert AMP-like functions by modulating the immune response. Aggregated or secreted α-syn can serve as a signaling molecule, activating microglia, dendritic cells, and other immune effectors through receptor-mediated pathways (24). This immunomodulatory activity may enhance pathogen clearance by stimulating cytokine release and adaptive immune activation. However, the same properties that enable α-syn to act as a defense molecule may also underlie its pathogenic potential. Excessive or prolonged induction of α-syn in response to infection may tip the balance toward misfolding and aggregation (25), thus linking its antimicrobial role with the initiation of neuroinflammatory and neurodegenerative cascades. In this sense, α-syn may represent a double-edged sword: protective against acute infection but potentially deleterious in the long term, contributing to synucleinopathy pathogenesis.

From an evolutionary perspective, the acquisition of antimicrobial peptide–like (AMP-like) properties by α-syn may reflect the selective pressures imposed by pathogens on the nervous system. Neurons, unlike many other cell types, are long-lived and irreplaceable, making their protection against microbial invasion particularly critical. It is therefore plausible that α-syn evolved as a multifunctional protein, combining roles in synaptic physiology with defense functions aimed at safeguarding neuronal integrity (26). Its structural plasticity, characterized by an intrinsically disordered state and the capacity to adopt amphipathic a-helical conformations (27), is reminiscent of classical AMPs, suggesting that its membrane-interacting properties may have been evolutionarily co-opted for both synaptic regulation and antimicrobial defense.

The evolutionary conservation of α-syn across vertebrate species further supports its dual functionality (28). Proteins with overlapping physiological and immunological functions often arise in contexts where host defense must be integrated with tissue-specific requirements. In the case of α-syn, its localization at presynaptic terminals positions it at a strategic interface between neuronal activity and potential pathogen entry points, particularly in peripheral sites such as the enteric nervous system and olfactory pathways, both recognized as portals for viral invasion. Thus, the AMP-like properties of α-syn can be interpreted as an adaptive trait, ensuring that neurons are equipped with a localized, inducible defense system. However, this same evolutionary strategy may carry inherent costs: mechanisms that enhance survival in the face of acute infection may predispose to maladaptive aggregation and neurodegeneration in aging organisms.

### α-Synuclein as an innate immune effector: evolving perspectives

Recent findings indicate that α-syn is not merely a neuronal protein but also plays active roles in the modulation of immune responses. Consistent with this view, α-syn has been shown to be required for normal immune function, acting as an alarmin-like mediator that promotes inflammatory responses and antigen-specific immunity through activation of macrophages and dendritic cells (29). At the level of innate immunity, extracellular and aggregated α-syn can function as a damage-associated molecular pattern (DAMP). These forms are recognized by pattern recognition receptors (PRRs), including toll-like receptors (TLR2, TLR4), expressed on microglia, macrophages, and dendritic cells (12, 30, 31). Their engagement initiates signaling cascades that drive pro-inflammatory cytokine production, reactive oxygen species release, and enhanced antigen presentation. Such activity positions α-syn as a potential sentinel molecule at the neuroimmune interface, contributing to the early orchestration of inflammatory responses. In the context of viral infection, this DAMP-like activity may amplify antiviral immune signaling, although excessive or sustained activation could favor inflammatory conditions permissive for aggregation-prone α-syn species.

Beyond innate mechanisms, α-syn also influences adaptive immunity. Misfolded or post-translationally modified variants of the protein can be internalized, processed, and presented via major histocompatibility complex (MHC) pathways, leading to T cell activation. Notably, early experimental studies showed that nitrated α-synuclein can generate neoantigenic epitopes capable of eliciting antigen-specific T cell and humoral responses, accompanied by enhanced MHC class II expression in draining lymph nodes. Adoptive transfer of T cells primed against nitrated α-syn further accelerated microglial activation and dopaminergic neurodegeneration *in vivo*, supporting a mechanistic link between oxidative α-syn modifications, loss of immune tolerance, and pathogenic adaptive immunity in parkinsonian models (32). Indeed, α-syn-derived epitopes have also been identified as immunogenic in patients with PD, where both CD4^+^ and CD8^+^ T cell reactivity have been observed (33). These findings suggest that α-syn not only bridges innate recognition with adaptive specificity but may also contribute to the breakdown of self-tolerance and the generation of autoreactive responses. Notably, these adaptive responses are best supported in the context of synucleinopathy and aggregate-associated inflammation, and their contribution to direct antiviral protection remains less clearly defined than α-syn-linked innate and interferon-associated mechanisms.

Several hypotheses have been advanced to explain these dual roles. A prevailing view is that α-syn functions as an inducible effector molecule, analogous to classical AMPs: upregulated in response to infection or inflammation, it may directly interfere with microbial viability or indirectly modulate host immunity. However, when persistently induced or inadequately cleared, α-syn may acquire pathogenic properties, acting as an autoantigen that drives chronic neuroinflammation and autoimmunity.

Taken together, these observations converge on the notion that α-syn represents a unique example of a neuronal protein with dual immunological functions: an AMP-like effector of innate defense and a potential autoantigen shaping adaptive immunity. While such properties may confer evolutionary advantages against acute infections, they also introduce vulnerabilities, as chronic or dysregulated immune engagement can favor protein aggregation, sustained inflammation, and ultimately the pathogenesis of synucleinopathies. Open questions remain regarding the precise triggers, regulatory checkpoints, and pathogen-specific contexts in which these functions are activated.

### Pathogen-triggered expression of α-synuclein: insights from experimental and translational evidence

Viral infections can influence α-syn expression and biology at multiple levels, beginning with transcriptional and translational regulation. Several studies have shown that viral challenge induces a rapid upregulation of α-syn, possibly as part of an innate protective response aimed at restricting viral replication or modulating host immune activation. This induction may be mediated by interferon-dependent pathways and other stress-responsive transcriptional programs, suggesting that α-syn could act as a stress-responsive mediator at the crossroad of antiviral defense and neuronal homeostasis (14). While transient increases in α-syn may be protective, prolonged dysregulation of its expression or clearance mechanisms could shift its biology toward pathogenic outcomes.

**Fig 1:**
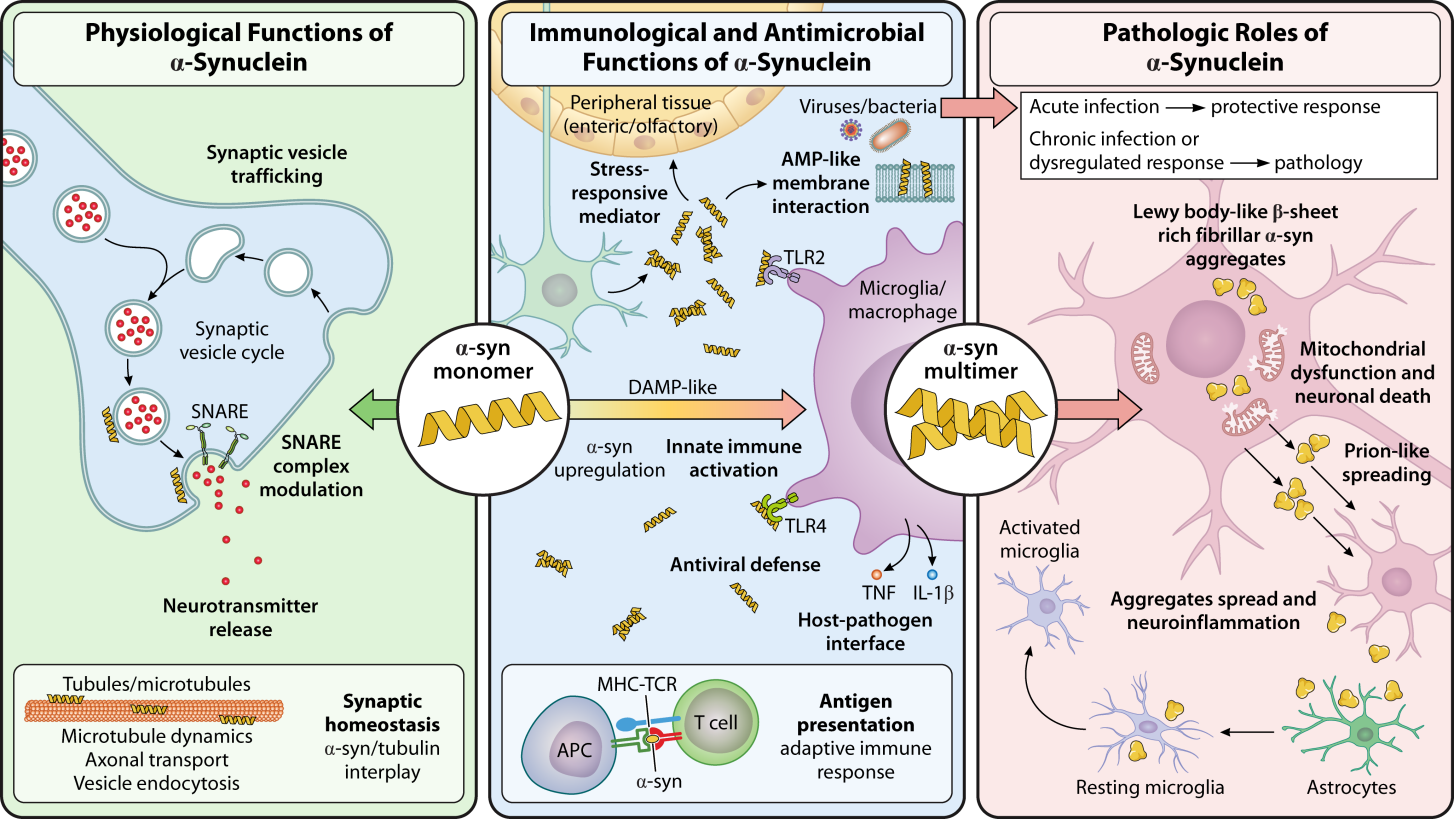
Context-dependent functions of α-synuclein across physiological, immunological, and pathological states. The biological roles of α-syn are highly context dependent. Under physiological conditions (left panel), α-syn localizes at presynaptic terminals, where it regulates synaptic vesicle trafficking, SNARE complex assembly, and contributes to neurotransmitter release and synaptic homeostasis. Additional roles include the regulation of microtubule dynamics, axonal transport, and vesicle endocytosis through interactions with tubulin and cytoskeletal components. Beyond the nervous system (central panel), α-syn functions as a stress-responsive mediator at host–pathogen interfaces, particularly in peripheral tissues. In this context, α-syn exhibits antimicrobial peptide (AMP)-like properties, interacts with microbial membranes, and can act as a damage-associated molecular pattern (DAMP), activating innate immune responses through pattern recognition receptors including TLR2 and TLR4 on microglia and macrophages. α-Syn upregulation may support antiviral defense and promote cytokine production, while also contributing to antigen processing and presentation via MHC I/II, with potential downstream engagement of CD8^+^ and CD4^+^ T cell responses. Under conditions of chronic infection, sustained inflammation, or impaired proteostatic control (right panel), α-syn undergoes pathological misfolding and aggregation into β-sheet-rich fibrillar assemblies. These aggregates disrupt mitochondrial function, promote neuronal dysfunction and death, and propagate in a prion-like manner across neural circuits. Persistent α-syn aggregation is associated with chronic neuroinflammation, characterized by microglial and astrocytic activation, ultimately contributing to the development and progression of synucleinopathies.

In addition to enhanced expression, viral infections can create a cellular environment that promotes α-syn aggregation (15). Viral replication and the associated inflammatory milieu perturb proteostatic networks by impairing autophagy, ubiquitin–proteasome pathways, and mitochondrial function, all of which are critical for α-syn clearance. Oxidative stress and the accumulation of viral proteins can further destabilize α-syn conformational equilibrium, fostering its transition from soluble monomers to oligomers and fibrils (34, 35). These aggregated species are neurotoxic and can amplify immune activation through engagement of pattern recognition receptors, thereby linking infection-driven proteostatic stress to chronic neuroinflammation (Fig. 1).

As anticipated, viral infections may facilitate the intercellular spread of α-syn aggregates, a phenomenon increasingly recognized as prion-like. Infected or stressed neurons can release misfolded α-syn into the extracellular space via exocytosis, vesicular secretion, or cell death (36). These pathogenic species may then be internalized by neighbouring neurons or immune cells, propagating aggregation in a templated fashion. Viral infections may accelerate this process by enhancing vesicular trafficking, increasing blood–brain barrier permeability, and amplifying inflammatory signaling, collectively fostering an environment conducive to α-syn dissemination (Fig. 1). This prion-like spread offers a mechanistic link between transient viral insults and the progressive, stereotyped pathology observed in PD and related synucleinopathies.

Building on this immunological and proteostatic framework, the following sections summarize virus-specific experimental models in which α-syn is modulated during infection, highlighting convergent mechanisms and context-dependent outcomes.

## α-SYNUCLEIN IN VIRAL INFECTION

### Cell culture studies: viral infections that alter α-synuclein

A substantial body of *in vitro* research shows that numerous neurotropic and systemic viruses can disturb α-syn homeostasis in different and sometimes opposing ways, influencing its abundance, aggregation, subcellular localization, post-translational patterns, and secretion. Work on Influenza A (H1N1) in human dopaminergic neurons reveals that infection rapidly drives α-syn aggregation without raising total protein levels, pointing to autophagy impairment—specifically, blocked autophagosome formation and reduced autophagic flux—as the main mechanism, an effect reversed by inhibiting viral replication (15). Flaviviruses, such as West Nile virus and Venezuelan equine encephalitis virus, increase α-syn levels and redistribute it to ER-derived membranes; loss-of-function studies show that neurons lacking α-syn support higher viral replication and exhibit more damage, indicating that α-syn contributes to an intrinsic antiviral defense linked to ER stress signaling (17). Studies of Japanese encephalitis virus further support a context-dependent protective role: neuronal α-syn overexpression suppresses viral replication, whereas conditions that heighten oxidative stress enhance vulnerability, suggesting that α-syn modulates redox balance during infection (18). In contrast, coxsackievirus B3 drives the formation of α-syn-positive, Lewy body-like inclusions enriched in damaged mitochondria and fibrillar α-syn by blocking late autophagy; here, α-syn overexpression accelerates inclusion formation and can even facilitate viral growth, underscoring that α-syn’s influence is virus-specific (37). For SARS-CoV-2, studies in human epithelial lung cell lines show a biphasic response—initial α-syn upregulation followed by a marked drop in synuclein alpha (*SNCA*) transcripts and protein—an effect moderated by interferon-β, with SNCA knockdown modestly increasing viral output. Complementary biophysical and co-culture work demonstrates that the viral nucleocapsid protein can directly accelerate α-syn fibrillization, while spike protein may enhance α-syn aggregation indirectly through microglia-derived cytokines and mitochondrial ROS, especially when neuronal α-syn is already elevated. Collectively, these *in vitro* systems reveal multiple virus-dependent routes—ranging from direct protein–protein interactions to immune-mediated and proteostasis-related mechanisms—through which viral infections reshape α-syn biology. Taken together, these *in vitro* findings provide strong evidence that viral infection can disrupt α-syn dynamics. Across cell systems, productive viral infection is sufficient to perturb α-syn homeostasis, most consistently by inducing α-syn and/or impairing its clearance (influenza, WNV, JEV, CVB3). Whether this shift is host-protective (restriction of flaviviruses via ER/IFN pathways) or host-detrimental (aggregation under autophagy blockade that can even aid picornaviral replication) appears virus- and pathway-specific. These patterns provide a mechanistic bridge between epidemiologic observations of post-infectious parkinsonism and α-syn pathology, while highlighting that the same protein can function as an inducible antiviral effector or as an aggregation-prone DAMP, depending on the virological and cellular context.

### Evidence from animal models

*In vivo* studies across multiple viral models demonstrate that diverse neurotropic agents can reshape α-syn biology through overlapping yet virus-specific pathways. Work on influenza viruses shows that neuroinvasive H5N1 infection in mice triggers sustained microglial activation and long-lasting neuronal α-syn phosphorylation and aggregation, while complementary H1N1 studies in both mouse brain and human dopaminergic neurons indicate that autophagy disruption, rather than increased SNCA transcription, drives α-syn accumulation, underscoring influenza’s ability to elevate α-syn levels, shift it toward phosphorylated α-synuclein at serine 129 (pSer129 α-syn), and impede its clearance—three levers that favor pathological α-syn species (15, 38). Phosphorylation at Ser129 is widely used as a marker of synucleinopathy-associated α-syn pathology, and its induction after infection is therefore commonly interpreted as a shift toward disease-relevant α-syn species. Mouse studies of WNV establish functional links between α-syn and antiviral defense, with knockouts showing higher brain viral loads, worse neuronal injury, and reduced survival, and with neurons upregulating α-syn following infection.

West Nile virus infection reveals a protective, neuron-intrinsic antiviral role for the protein, although definitive murine evidence of aggregate formation remains incomplete, despite human case reports and related arboviral models showing pSer129-positive, proteinase K-resistant deposits (17, 39, 40). In this case, the lack of rigorous data on aggregation in WNV represents a significant gap in confirming the link to synucleinopathic pathology.

For HSV-1, although mouse studies primarily highlight amyloid-driven pathology and glial inflammation, with accelerated Aβ deposition and cognitive decline, direct *in vivo* evidence of α-syn changes remains limited and often confounded by severe encephalitic models. However, *in vitro* systems, mainly neuronal cell lines and organoids, suggest that HSV-1 can alter α-syn expression and that the strong glial-inflammatory milieu and autophagy perturbations should be permissive for α-syn dysregulation. Yet, rigorous murine data sets showing region-resolved increases in α-syn (total or pSer129) after HSV-1 infection remain limited and often confounded by severe encephalitic paradigms. Biological plausibility is high, given shared pathways with influenza and arboviruses, but definitive *in vivo* evidence for α-syn modulation by HSV-1 in mouse brain still requires standardized, aggregate-selective assays and time courses that capture subacute windows beyond the acute encephalitic phase (41, 42).

In contrast, Western equine encephalitis virus (WEEV) produces clear synucleinopathic signatures: sublethal intranasal infection yields long-lasting, proteinase K-resistant pSer129 α-syn inclusions across limbic and midbrain regions, accompanied by dopaminergic neuron loss and gliosis driven by astrocytic NF-κB signaling (39, 43). Deletion of this signaling mitigates α-syn aggregation, gliosis, and neuron loss, arguing that glia-driven inflammation is sufficient to drive α-syn pathology after encephalitic infection.

Coxsackievirus B3 (CVB3) also promotes α-syn aggregation in midbrain neurons and, in α-syn-transgenic mice, enhances viral growth and accelerates degeneration of neurons in substantia nigra, consistent with its blockade of late autophagy creating conditions favorable for inclusion formation (37).

Finally, SARS-CoV-2 worsens synucleinopathy in hACE2-transgenic mice pre-seeded with α-syn fibrils, amplifying pSer129 α-syn accumulation, gliosis, and dopaminergic impairment long after viral clearance (44), while spike S1 alone can induce α-syn aggregation and microglial activation in rodents through cytokine- and ROS-dependent pathways that are partially mitigated by metformin. While this is not a full infection model, it is still *in vivo* evidence that a viral protein can drive α-syn pathology via neuroimmune routes (45). The potential of metformin as a modulator represents an intriguing therapeutic avenue for further exploration.

Collectively, these models provide strong functional evidence for the role of viral infections in modulating α-syn, with both direct impacts (aggregation, post-translational modifications) and indirect effects (inflammation, proteostatic stress). The involvement of glial inflammatory mechanisms emerges as a crucial factor. There are still gaps, especially regarding precise quantification of aggregates in some models (WNV, HSV-1) and the translation to natural infection conditions. The diversity of responses and mechanisms depending on the virus highlights the complexity of the system and the need for more specific and extended temporal models.

## POTENTIAL MECHANISMS LINKING VIRAL INFECTIONS AND α-SYNUCLEIN BIOLOGY

As reported, a growing body of evidence suggests a compelling link between viral infections and the regulation, misfolding, and aggregation of α-syn. Several mechanistic pathways have been proposed to explain how viruses might influence α-syn biology, either directly by altering its expression and processing or indirectly through inflammation and cellular stress.

### Alteration of α-synuclein expression or processing

A number of reports suggest that viral infections can upregulate α-syn expression as part of the innate antiviral response, serving as a cellular defense mechanism. This increased expression may, however, have unintended consequences, as it can promote the aggregation of α-syn into toxic oligomers and fibrils. Additionally, viral infections can induce post-translational modifications of α-syn, including phosphorylation, that further enhance its propensity to misfold and aggregate. These changes not only exacerbate α-syn pathology but may also impair normal cellular functions, contributing to neuroinflammation, synaptic dysfunction, and neuronal death.

Beatman et al. reported that WNV infection induces a robust increase in α-syn levels in mouse primary neurons and in tissue samples from patients with acute encephalitis (17). This increase was associated with a protective response, as α-syn, in turn, inhibited WNV growth and disease in the CNS, likely limiting viral replication through modulation of synaptic vesicle dynamics or by interfering with viral RNA processing (17). According to this model, virus-induced α-syn accumulates at ER-derived membranes, modulates endoplasmic reticulum stress signaling, and restricts viral replication, propagation, and CNS injury—ultimately reinforcing its function as an antiviral effector.

Evidence has also shown that SARS-CoV-2 spike (S1) and nucleocapsid (N) proteins induced increased expression of α-syn *in vitro* in HEK293 cells (46). Furthermore, in these cells, α-syn exhibited a tendency to aggregate in the perinuclear region, where it was found to colocalize and likely interact with SARS-CoV-2 proteins, thus indicating that SARS-CoV-2 proteins can directly affect α-syn biology (47). Indeed, there is emerging evidence suggesting a potential link between COVID-19 and either the rapid progression of PD or the development of temporary PD-like symptoms (48–51). In line with this notion, the ability of the S1 protein to promote pathological modifications of α-syn, in particular the phosphorylation at serine 129 (pSer129), which is a hallmark of synucleinopathies present in Lewy bodies, likely reflects the importance of virus-induced molecular alterations in the initiation or acceleration of neurodegenerative processes (52, 53). *In vitro* studies revealed that S1 interacts with α-syn, promoting its phosphorylation and aggregation (46, 54, 55), with inflammation and oxidative stress potentially contributing to this process (47). The S1 subunit of the SARS-CoV-2 spike protein, which contains the fragment S194, has been shown to interact with α-syn, facilitating the formation of protein aggregates capable of causing synaptic impairment and cytotoxicity in a cellular model of synucleinopathy (55, 56). In particular, in HEK293 cells, the S1 domain interacted with α-syn and favored its aggregation. Also, the S1 domain induced mitochondrial dysfunction, oxidative stress, and cytotoxicity. Remarkably, proteolytic digestion of the spike protein by the enzyme neutrophil elastase, which is associated with the host inflammatory response, resulted in the formation of fibrillogenic fragments *in vitro* (57). Similarly, SARS-CoV-2 N protein was found to interact with α-syn, accelerating its fibrillation (54). Recent evidence indicates that not only the isolated S and N proteins of SARS-CoV-2 can promote α-syn aggregation, but also that the spatial organization of the spike protein in a corona-like arrangement on the viral envelope may play a critical role in accelerating amyloid fibril formation, potentially contributing to the pathogenesis of Parkinson’s disease (58).

A few additional viruses have been suggested to influence α-syn post-translational modifications and aggregation, although they are not consistently addressed in discussions of infection-related synucleinopathy. Hepatitis C virus (HCV) infection has been linked to systemic and neural inflammation, oxidative stress, and mitochondrial impairment—conditions that may promote biochemical modifications of α-syn and increase its propensity to misfold and aggregate. Indeed, HCV has been reported to upregulate SLC30A2 gene expression, leading to zinc ion dysregulation, which has been associated with arteriosclerosis and may facilitate α-syn aggregation (59). Epstein–Barr virus (EBV), a widespread herpesvirus that establishes persistent latent infection, can alter immune signaling and inflammatory pathways, potentially affecting cellular environments that regulate protein folding and neuronal homeostasis. In particular, anti-EBV-LMP1 antibodies could cross-react with a defined α-syn epitope, thus inducing its aggregation (60). In addition, human pegivirus (HPgV) has received limited attention in neurodegenerative research, but its recognized ability to modulate immune responses suggests that it could indirectly influence processes associated with α-synuclein regulation (61). Although current evidence remains largely indirect, these viruses highlight the broader possibility that chronic or systemic viral infections may contribute to molecular conditions favoring α-syn modification and aggregation.

Other than SARS-CoV-2, influenza A H1N1 virus also induced aggregation of endogenous α-syn in differentiated, dopaminergic, neuron-like Lund human mesencephalon (LUHMES) cells, as well as in neurons connected to the olfactory bulb (15). In this setting, H1N1 inhibited autophagosome formation and impaired autophagic flux, thereby favoring α-syn aggregation. Notably, α-syn aggregates can impair autophagy (62), creating a self-perpetuating cycle in which autophagy inhibition further promotes α-syn aggregation. Similarly, the virion-associated HIV-1 viral protein R (Vpr) has been shown to induce the accumulation of α-syn aggregates and disrupt mitochondrial axonal transport in neurons by impairing the autophagy–lysosomal degradation pathway and disrupting other key processes required for the clearance of protein aggregates, including SNAPIN (63). Indeed, clinical studies suggest that elderly individuals living with HIV-1 may experience motor dysfunction symptoms, including bradykinesia, rigidity, postural instability, gait abnormalities, and tremors, which could be due to the accumulation of aggregated α-syn in neurons of the basal ganglia (64). However, HIV-associated neurocognitive disorders and the persistence of viral reservoirs in the brain remain major challenges, and despite their clinical significance, the mechanisms underlying HIV-1 entry and replication within the CNS are still not fully understood. Although α-syn has been implicated in antiviral defense mechanisms, its role is not exclusively protective. It has been shown that amyloid fibrils formed by α-syn can boost HIV-1 attachment, fusion, and replication in human T cells, macrophages, and microglia (65). Fibrils did not circumvent the requirement for CD4 and coreceptor for HIV-1 infection but reduce the threshold levels of these receptors required for viral infection. *In vitro* studies showed that α-syn released from cells oligomerized and facilitated HIV-1 infection, as α-syn fibrils enabled R5-tropic HIV-1 to infect U373-MAGI cells despite the absence of detectable CCR5 expression, thereby facilitating viral entry and potentially contributing to HIV-1 dissemination within the brain (65). Notably, studies have reported that α-syn is upregulated during immune responses, particularly in the substantia nigra of individuals infected with HIV, and that the accumulation of fibrils may result from impaired lysosomal clearance induced by Vpr (9, 63, 66). Another recent study linked α-syn to autophagy. It was found that infection of neurons with CVB3, a single-stranded RNA virus belonging to the *Picornaviridae* family of viruses in the genus *Enterovirus*, induced the formation of α-syn-associated inclusion bodies, containing clustered organelles including damaged mitochondria with α-syn fibrils (37). In this system, inclusion bodies formed mainly because CVB3 inhibited the late stage of autophagy. Interestingly, brains of mice infected with CVB3 also harbored α-syn aggregates within midbrain neurons, thus suggesting a possible mechanism of Lewy body formation associated with CVB3 infection (37).

In summary, the vast majority of the studies reporting a mechanistic association between viral infections and α-syn biology involve RNA viruses (67). These include influenza A virus, enteroviruses, and more recently, SARS-CoV-2, all of which have been shown to influence α-syn expression, aggregation, or propagation. RNA viruses are particularly relevant due to their high mutation rates, strong immunogenicity, and frequent neurotropism, which allows them to infect cells within the central and peripheral nervous systems. Their ability to trigger robust inflammatory responses, disrupt proteostasis pathways such as autophagy and the ubiquitin–proteasome system, and interact directly or indirectly with α-syn positions them as key players in the investigation of environmental factors contributing to synucleinopathies. Conversely, it would also be of interest to further investigate the potential role of α-syn as an active component of the innate immune response to these viruses, raising the possibility that its initial protective function could, under dysregulated conditions, contribute to pathology.

### Increase of α-synuclein through immune activation and/or inflammation

Viral infections can indirectly induce α-syn upregulation or modifications through immune activation and/or inflammation. Several studies have demonstrated increased α-syn expression following inflammatory challenges to the CNS, the gastrointestinal nervous system, or the peripheral nervous system (PNS) (68–70). Moreover, the presence of early inflammatory responses, observed both in experimental models and in Parkinson’s disease patients prior to the deposition and propagation of α-syn, suggests a mechanistic link between inflammation and α-syn pathology.

Initial findings demonstrated that intranasal infection of mice with a neurotropic strain of the H5N1 influenza virus allows the virus to spread from the PNS to the CNS, where it subsequently triggers an innate immune response (38). In addition, this infection has been shown to induce not only parkinsonian symptoms but also a marked increase in α-syn phosphorylation and aggregation, likely contributing to the degeneration of nigral dopaminergic neurons (38, 71). Other neuroinvasive infections can cause post-encephalitic parkinsonism, potentially through loss of dopaminergic neurons and neuroinflammation (39). It was reported that infection with mosquito-borne alphaviruses, such as Western equine encephalitis virus (WEEV), causes activation of microglia and astrocytes, loss of dopaminergic neurons in the SNpc, and neurobehavioral abnormalities early after viral insult (39). Notably, abundant proteinase K-resistant aggregates of pSer129 α-syn were observed in the entorhinal cortex, hippocampus, and basal midbrain. After viral infection, astrocytes and microglia can acquire a persistent neurotoxic and pro-inflammatory phenotype which may aid in controlling the infection but also contribute to neuronal degeneration (72). Supporting this notion, previous studies have shown that WEEV infection in CD-1 mice led to elevated expression of cytokines and chemokines, such as CCL2 and CCL10, in surviving animals (73). Moreover, neuroinflammatory activation of glial cells following infections may work in tandem with neuronal oxidative stress and mitochondrial dysfunction to promote protein misfolding and α-syn aggregation.

Both oxidative stress and mitochondrial impairment, *per se*, have long been implicated in α-syn alterations. Concerning oxidative stress, large oscillations in intracellular calcium concentration within vulnerable nigral neurons lead to mitochondrial oxidant stress, dopamine oxidation, lysosomal dysfunction, and α-syn accumulation (74, 75). As intracellular α-syn levels rise, a vicious cycle may be triggered, in which the excess protein promotes aggregate formation, and these aggregates, in turn, intensify oxidative stress (76, 77). At least in neurons, oxidative stress is strongly interconnected with mitochondrial dysfunction and α-syn aggregation. Indeed, α-syn aggregates interact transiently and dynamically with mitochondria, causing mitochondrial depolarization, reduced ATP production, mitochondrial fragmentation, and degradation through cardiolipin externalization–dependent mitophagy (78).

In mice, intravenous SARS-CoV-2 spike protein induces neuroinflammation and α-syn accumulation in brain regions relevant to PD (47). Neuroinflammation could be one of the main factors contributing to neurological symptoms of COVID-19 and, if persistent, may explain the long-term neurological aspects of post-COVID-19 (79–81). The capacity of the S1 protein to induce α-syn aggregation has been demonstrated in both *in vitro* systems and rodent models, where neuroinflammation appears to be a critical prerequisite for this process (45). Indeed, in rat brain administered with S1, α-syn aggregation in the SNpc region correlated with the expression of ionized calcium-binding adapter molecule 1 (Iba1), which is a marker of activated microglia. Consistent with this, conditioned media from S1-stimulated BV2 microglial cells elevated α-syn monomer levels, enhanced its phosphorylation at Ser129, and increased the accumulation of aggregated forms through a pro-inflammatory mechanism (82). Additionally, S1 can directly promote α-syn aggregation by elevating mitochondrial reactive oxygen species (ROS), particularly when present at sufficient levels within the cell. Finally, S1 acts synergistically with the dopaminergic neurotoxin 1-methyl-4-phenylpyridinium (MPP+), leading to heightened ROS production, mitochondrial damage, and decreased viability of dopaminergic neurons (45, 83).

Stolzenberg et al. showed that α-syn expression in the enteric neurites of the upper gastrointestinal tract in pediatric patients was positively correlated with the severity of both acute and chronic inflammation triggered by norovirus in the intestinal wall (69). Building on these observations, subsequent studies showed that intraperitoneal administration of inflammatory agents, such as bacterial peptidoglycan and thioglycolate, can induce α-syn production within the inflammatory environment of the peritoneal cavity, as well as promote its expression in neuronal tissues of the diaphragm and colon (29). In turn, α-syn induced the expression of pro-inflammatory cytokines, including TNF and IL-12p70, by dendritic cells and macrophages, thus indicating that a loop perpetuating inflammation could be generated and maintained. In line with this notion, multiple lines of evidence suggest that α-syn is essential for sustaining the full expression profile of interferon-stimulated genes (ISGs) in brain tissue following WNV infection. Indeed, studies in mouse models have shown that the expression of *Oas1b*, *Irf9*, *Trim25*, and *Tgtp1*, among others, was reduced in the brain tissue of WNV-infected α-syn knockout (KO) mice compared to wild-type (WT) mice (40). Upon type I IFN stimuli, α-syn induced transcription of ISGs by interacting with pSTAT2 to favor the nuclear translocation of the pSTAT1–pSTAT2 heterodimer (14, 40). Conversely, no differences were observed between WT and KO mice in the expression of TNF, IL-6, IFN-α, IFN-β following WNV infection (17).

**Fig 2:**
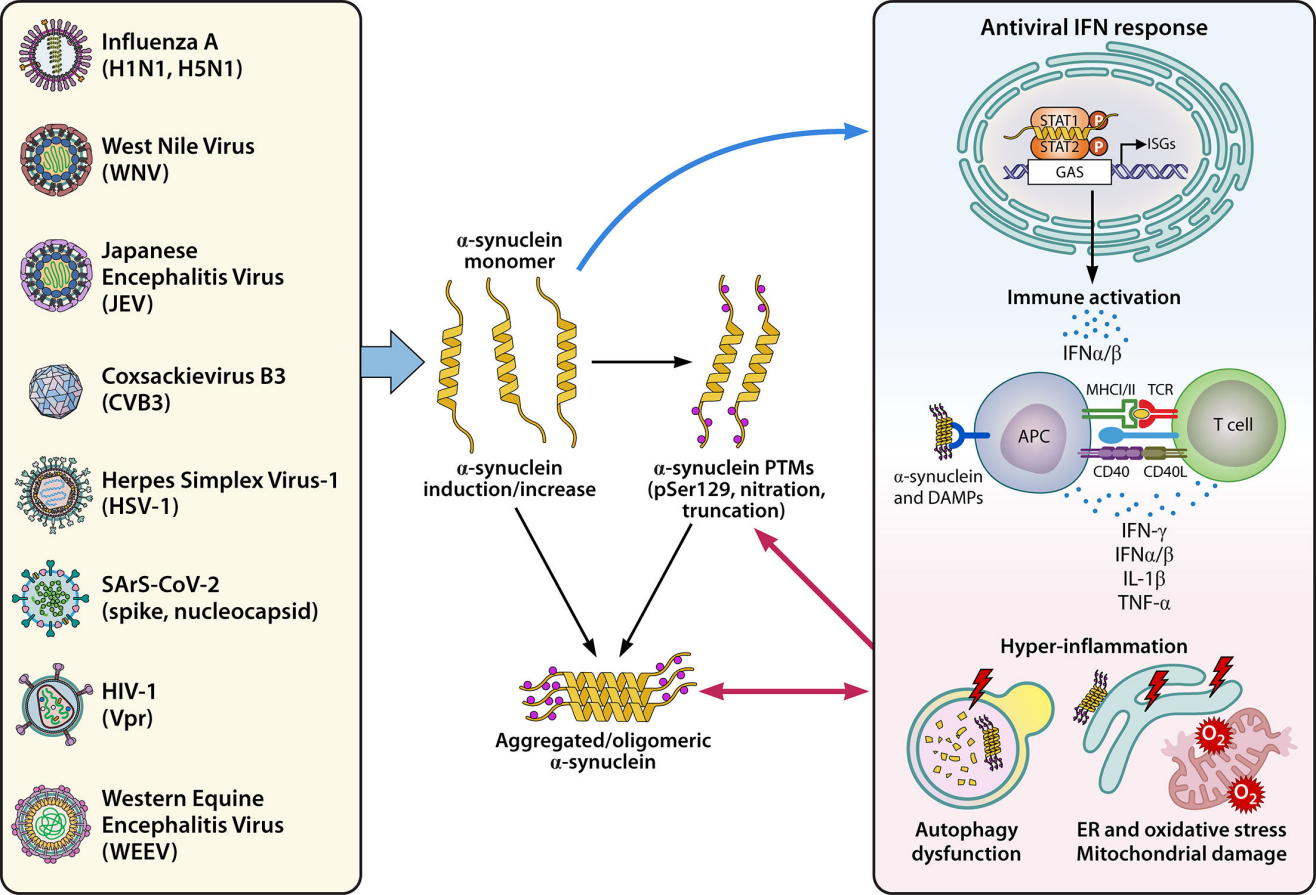
Virus-induced pathways linking α-synuclein to antiviral responses and aggregation-prone states. Viral infection can alter α-synuclein/SNCA expression and localization through interferon (IFN)-dependent and stress-responsive programs, supporting antiviral defense via activation of STAT2/ISG-associated pathways and immune activation/antigen presentation. In parallel, viral replication and inflammation can disrupt cell proteostasis (autophagy–lysosomal and ubiquitin–proteasome pathways), induce ER stress, oxidative stress, and mitochondrial injury, thereby favoring post-translational α-syn modifications (PTMs, including pSer129, nitration, and truncation) and promoting aggregation-prone species. Aggregated/oligomeric α-synuclein species further amplify inflammatory signaling, impair cell clearing systems, and exacerbate mitochondrial damage and ER/oxidative stress, establishing a self-reinforcing cycle of α-synuclein aggregation and cellular dysfunction, which can be deleterious for neuronal cells. For clarity, the figure separates (top) interferon-linked antiviral pathways from (bottom) proteostasis/inflammation-driven aggregation pathways.

Overall, these findings underscore α-syn as a mediator at the intersection of antiviral signaling and inflammation (Fig. 2). In some contexts, α-syn supports antiviral programs, particularly through interferon-dependent transcriptional responses and ISG maintenance (14, 38). In parallel, viral-driven inflammatory stress and impaired clearance pathways can promote pSer129 α-syn accumulation and aggregation, which may then amplify innate immune activation through PRR engagement and glial responses. Thus, antiviral restriction mechanisms and inflammatory responses to aggregation-prone α-syn species should be considered related but temporally and mechanistically distinct processes.

### Cross-seeding

Numerous studies have indicated that certain viral proteins may promote the formation of α-syn aggregates, either directly or through indirect mechanisms (15, 54, 55). In the aforementioned cases, viral proteins did not promote α-syn aggregation by catalyzing the formation of an aggregation-prone nucleus. Instead, they co-aggregate with α-syn, leading to the formation of composite fibrils in which the viral protein often becomes an integral structural component. Alternatively, they may disrupt cellular pathways such as autophagy, thereby creating conditions that favor α-syn accumulation and subsequent aggregation.

Recent studies have investigated the cross-seeding phenomenon, in which aggregates or fibrils formed by one protein can promote or accelerate the aggregation of another, often structurally similar, protein. It was found that the fibrillogenic spike fragment 194–203 (S194) promoted α-syn monomer fibrillation in a concentration-dependent manner, leading to aggregates with a greater ability to induce lipid vesicle leakage and neuroblastoma cell toxicity compared to either α-syn or S194 alone (84). Bi-dimensional NMR indicated that S194 fibrils caused a greater perturbation in both the N-terminal region (19–68) and the hydrophobic central domain (71–95) of the α-syn monomer, which is supported by protein-peptide docking and molecular dynamics simulations (84). This means that SARS-CoV-2 infection could accelerate or trigger neurodegenerative diseases linked to protein amyloidosis. However, *in vivo* studies should uncover whether fragments of the S protein can drive pathological cross-seeding with α-syn and contribute to long-term neurological consequences.

Considering these multifaceted and context-dependent effects, Table 1 summarizes the current experimental evidence supporting α-synuclein’s involvement in antiviral defense, immune activation, antigen presentation, and aggregation-prone pathways across central and peripheral systems.

**TABLE 1:** Context-dependent immune and antiviral functions of α-synuclein derived from experimental and mechanistic evidence^*a*^

Biological context	Mechanism/pathway involved	Experimental observations described	Functional consequence	References
Antimicrobial peptide–like activity	Amphipathic N-terminal domain enables insertion into lipid bilayers	Direct antimicrobial activity against bacterial and viral pathogens	Membrane destabilization; impairment of microbial viability; contribution to host defense	(17, 18, 22, 23)
Innate immune activation (extracellular/aggregated α-syn)	DAMP-like signaling via PRRs (TLR2, TLR4) on microglia, macrophages, dendritic cells	Engagement of PRRs induces pro-inflammatory cytokine production, ROS release, enhanced antigen presentation	Amplification of innate immune responses at the neuroimmune interface	(12, 24, 30, 31)
Adaptive immune activation	Processing and presentation via MHC pathways	α-syn-derived epitopes identified as immunogenic; CD4^+^ and CD8^+^ T cell reactivity observed	T cell activation; potential autoreactive responses	(33)
Enteric nervous system/mucosal interface	Inflammation-associated upregulation	Increased α-syn expression in enteric neurites correlates with severity of intestinal inflammation (e.g., norovirus)	Link between mucosal inflammation and neuronal stress responses	(69)
Interferon-dependent antiviral responses	Interaction with pSTAT2 promoting nuclear translocation of pSTAT1–pSTAT2 heterodimer	Reduced ISG expression (*Oas1b*, *Irf9*, *Trim25*, *Tgtp1*) in α-syn KO mice following WNV infection	Sustained ISG transcription; support of antiviral defense	(14, 40)
Viral infection–induced upregulation	Interferon-dependent and stress-responsive transcriptional programs	Rapid α-syn induction upon viral challenge	Potential restriction of viral replication; modulation of immune activation	(14)
Proteostasis impairment during viral infection	Autophagy–lysosomal and ubiquitin–proteasome dysfunction; mitochondrial injury	Influenza H1N1, CVB3 and other RNA viruses impair autophagic flux; α-syn aggregation promoted	Transition from soluble monomers to oligomers/fibrils; aggregation-prone state	(15, 37)
Direct viral protein interaction (SARS-CoV-2)	Spike (S1) and nucleocapsid (N) interaction; post-translational modifications (pSer129)	Increased α-syn expression and aggregation; accelerated fibrillization; ROS-dependent aggregation	Enhanced aggregation; potential contribution to neuroinflammation	(46–58)
HIV-1 infection	Vpr-mediated impairment of autophagy–lysosomal degradation; SNAPIN disruption	Accumulation of α-syn aggregates; α-syn fibrils facilitate HIV-1 attachment and replication	Context-dependent effects; potential facilitation of viral dissemination	(63–66)
Neuroinvasive viral infection (H5N1, WEEV)	Microglial and astrocytic activation; NF-κB signaling; oxidative stress	Increased α-syn phosphorylation (pSer129), proteinase K-resistant aggregates, dopaminergic neuron loss	Neuroinflammation-associated aggregation; synucleinopathic features	(38, 39, 43, 71–73)
Oxidative stress and mitochondrial dysfunction	ROS production; mitochondrial depolarization; impaired mitophagy	α-syn aggregates interact with mitochondria; promote ATP reduction and fragmentation	Vicious cycle of oxidative stress and aggregation; neuronal vulnerability	(74–78)
Cross-seeding mechanisms	Co-aggregation with viral protein fragments (spike S194–203)	Concentration-dependent acceleration of α-syn fibrillation; enhanced membrane leakage and cytotoxicity	Potential trigger/acceleration of protein amyloidosis	(84)

^*a*^ Summary of experimental evidence indicating that α-syn acts as an antimicrobial peptide-like molecule, a modulator of innate and adaptive immunity, and a context-dependent antiviral effector. Viral infections induce α-syn upregulation through interferon-dependent and stress-responsive pathways, while proteostatic disruption, inflammation, and direct viral protein interactions may shift α-syn toward aggregation-prone and neurotoxic states. These dual properties support a framework in which α-syn contributes to host defense yet may also promote chronic neuroinflammation and synucleinopathic pathology under sustained stress conditions.

## CONCLUDING REMARKS

Collectively, the available evidence highlights a multifaceted relationship between viral infections and α-syn biology, encompassing direct molecular interactions, virus-induced cellular stress responses, and immune-mediated mechanisms. Viruses can influence α-syn at multiple levels, by upregulating its expression, altering its post-translational modification profile, disrupting its clearance, or physically interacting with it to promote aggregation. In particular, RNA viruses, such as SARS-CoV-2, influenza A, HIV-1, and enteroviruses, have emerged as key candidates capable of modulating α-syn homeostasis, potentially contributing to the initiation or acceleration of synucleinopathies. Moreover, α-syn itself may play an active role in the host antiviral defense, although this protective function can become detrimental under chronic or dysregulated conditions. Viral hijacking of neuronal transport systems, interference with autophagic flux, and cross-seeding between viral proteins and α-syn aggregates all represent plausible mechanisms linking infection to neurodegeneration. Understanding the interplay between infections and the dual nature of α-syn is crucial for managing and treating patients with pre-existing neurological conditions who contract COVID-19 or other diseases associated with the aforementioned infections. A key implication is that α-syn should be considered within a systemic immuno-proteostatic framework, where virus-induced perturbations may reverberate across peripheral immune and non-neuronal tissues as well, potentially intersecting with disease connections that extend beyond classical synucleinopathies.

While compelling, these observations largely arise from *in vitro* systems or animal models, and future research must clarify their relevance to human disease. In particular, *in vivo* studies are essential to determine whether virus-induced α-syn alterations represent a compensatory, potentially protective response, or whether they causally contribute to the development or progression of synucleinopathies and other neurological disorders. Several viruses have been proposed as potential contributors to the development of parkinsonism and PD; however, the nature and significance of this association remain controversial (19, 51).

Understanding these complex interactions may open new avenues for therapeutic strategies aimed at modulating host–virus interactions, controlling neuroinflammation, or preventing the misfolding and spread of α-syn in the context of infection-triggered neurodegeneration.

## Data Availability

This review summarizes key developments in virology research relevant to α-synuclein biology, based exclusively on evidence from peer-reviewed, published studies; no unpublished data are included.
